# Comparison of bispectral index and patient state index as measures of sedation depth during surgeries using remimazolam tosilate

**DOI:** 10.1186/s12871-023-02172-3

**Published:** 2023-06-15

**Authors:** Tang-yuan-meng Zhao, Di Chen, Zhi-xin Xu, Huan-liang Wang, Hu Sun

**Affiliations:** The Second Affiliated Hospital of Hainan Medical University, Hai Kou, China

**Keywords:** Remimazolam, BIS, PSI, Pearson’s correlation coefficient analysis

## Abstract

**Background:**

The Bispectral Index (BIS) and the Patient State Index (PSI) are commonly used measures to assess intraoperative sedation depth. However, model differences lead to different results, which in turn interferes with clinicians’ judgment on the depth of anesthesia. Remimazolam tosilate (RT) for injection is a new benzodiazepine used in sedation. In its clinical application, there are few effective indicators for sedation depth monitoring. To close this gap, this study aims to compare BIS and PSI in measuring the sensitivity and specificity of intraoperative RT and to explore the safety of RT for intraspinal anesthesia in elderly patients.

**Methods:**

This study included 40 patients undergoing elective electro-prostatectomy with intraspinal anesthesia, who were monitored by BIS and PSI simultaneously during operation. Remimazolam tosylate 0.1 mg/kg was intravenously administered after the intraspinal anesthesia when patients were in a completely painless status. Then BIS, PSI, the Modified Observer’s Assessment of Alertness and Sedation (MOAA/S) scores and vital signs were observed and recorded per minute for 10 min. Pearson’s correlation analysis and linear regression model were used to compare BIS and PSI sedation scores, and to test their associations with the MOAA/S score, respectively. ROC curves were drawn to compare the sensitivity and specificity of BIS and PSI. Changes of vital signs were presented as mean ± standard deviation. Perioperative liver and kidney function indicators were analyzed using a paired t-test to evaluate the safety of RT for intraspinal anesthesia in the elderly patients.

**Results:**

According to Pearson’s correlation analysis, a significant (*P* < 0.01) correlation between BIS and PSI was found when used to monitor intraoperative sedation of RT (*r* = 0.796). Significant associations between BIS and MOAA/S (*r* = 0.568, *P* < 0.01), and between PSI and MOAA/S (*r* = 0.390, *P* < 0.01) were also found. The areas under the ROC curves of BIS and PSI were 0.801 ± 0.022 and 0.734 ± 0.026, respectively, suggesting that both measures may predict patients’ state of consciousness and BIS was more accurate than PSI. Vital signs remained stable throughout the study. No abnormal changes of clinical significance were found based on laboratory test results of liver and kidney function.

**Conclusion:**

BIS and PSI are strongly associated for monitoring the sedation of RT intraoperatively. Both methods can accurately reflect sedation depth. According to correlation analyses with MOAA/S scale and ROC curves, BIS is more accurate than PSI during such intraoperative monitoring. In addition, RT can be safely used in elderly patients under intraspinal anesthesia for supportive sedation, with stable vital signs and sound kidney and liver safety profiles.

**Trial registration:**

http://www.chictr.org.cn (ChiCTR2100051912).

**Supplementary Information:**

The online version contains supplementary material available at 10.1186/s12871-023-02172-3.

## Background

The depth of anesthesia may greatly impact patients’ physiological state. An appropriate depth may reduce the incidence of intraoperative adverse events and postoperative complications. Insufficient depth of anesthesia will lead to intraoperative pain, abnormal sympathetic innervation, and even intraoperative awareness; while excessive anesthesia, on the other hand, will directly inhibit patients’ cardiac and cerebrovascular functions, delay their recovery, and increase their risks of postoperative complications such as respiratory depression. In particular, the sedation depth for elderly patients should be appropriately and accurately monitored owing to declined physiological functions in this population [1–3].

The Bispectral Index (BIS), a sedation monitoring index originally developed for propofol, has been widely applied in assessing intraoperative sedation in clinical practices. Although more commonly used in surgeries under general anesthesia, with the advancement of clinical research in anesthesiology, BIS also has a vital role in intraoperative sedation monitoring to guide surgical and anesthetic procedures. According to previous studies, the sensitivity of BIS for benzodiazepines such as midazolam remains controversial, and further research is needed [4–6].

The Patient State Index (PSI; Physiometrix Inc., North Billerica, MA, USA), in contrast, is calculated via a proprietary algorithm by a high-resolution 4-channel electroencephalograph (EEG) monitor. EEG parameters of different consciousness states are calculated and processed to build a complete database. Having been validated in a great number of trials, PSI can reflect and predict variations of consciousness status caused by anesthetics. Unlike BIS, PSI is built on statistical processing of EEG data, independent from medication and chemical reagent. As such, clinical sedation assessment using these two measures may yield different results [7–9].

Remimazolam tosilate (RT) for injection is a novel ultra-short-acting benzodiazepine used in sedation with a rapid onset. It is metabolized by tissue esterases and liver carboxyesterases, producing little impact on liver and kidney function, and thus has been widely applied in clinical anesthesia and sedation [10, 11]. Due to the decline of cardiopulmonary and neurological functions, elderly patients feature: 1. decreased liver and kidney function, increased sensitivity to anesthetic drugs, increased susceptibility to excessive sedation, and obvious changes in anesthesia monitoring index; 2. autonomic insufficiency, abnormal vascular elasticity and tension [1, 12, 13]. These features render them more prone to cardiopulmonary, hepatic and renal dysfunction after sedation. Therefore, our study aims to test the correlation between BIS and PSI as measures of anesthesia depth in elderly male patients undergoing assisted sedation with RT, and to assess their respective association with conventional MOAA/S scores. Secondary endpoints include commonly used clinical and laboratory test indicators to evaluate the safety of RT.

## Methods

### Study design with ethics and registration

This is a single-arm, single-center, prospective clinical trial, which was approved by the Institutional Review Board of the Second Affiliated Hospital of Hainan Medical College (Approval number: reference number 2021–024-02, 20/5/2021) and was registered on http://www.chinadrugtrials.org.cn/ (Registration number: ChiCTR2100051912, 9/10/2021). After obtaining the ethics approval documents, we recruited patients who were scheduled to undergo electro-prostatectomy with intraspinal anesthesia in the Second Affiliated Hospital of Hainan Medical College. All participants signed an informed consent.

### Patient inclusion and exclusion criteria

Patients aged 65–80 years were enrolled. An anesthesiologist conducted a pre-anesthesia assessment for patients one day before the operation to evaluate their physiological status and eligibility. Participants with an American Society of Anesthesiologists (ASA) physical status I or II and a BMI between 18–25 kg/m^2^ were included. Patients with: (1) contraindications to intraspinal anesthesia, including abnormal coagulation function, abnormal spinal structure, and infection at the puncture site; (2) benzodiazepine allergy; difficult airways as assessed by an anesthesiologist; (3) previously diagnosed hypertension, coronary heart disease and other cardiopulmonary dysfunction; (4) confirmed diagnosis of mental system disorder; (5) drug, alcohol and other psychotropic substance abuse; (6) abnormal laboratory test levels of liver and kidney function indicators; (7) abnormal heart and lung structure found on imaging exams were excluded. Patients withdrew from the study when the analgesia was insufficient, or the level of the intraspinal anesthesia was too high, or serious complications of intraspinal anesthesia occurred, including decreased blood pressure, slow heart rate, and respiratory depression.

### Perioperative management

On the day of the operation, an anesthesiologist prepared for the procedure, checked the performance of the anesthesia machine and monitors, tested functions of both the BIS and the EEG monitoring equipment, and prepared the combined spinal-epidural puncture package. RT((developed by Jiangsu Hengrui Medicine Co. Ltd., China, 201031AK, YBH03052019)for injection was diluted with 0.9% sodium chloride to a concentration of 0.5 mg/ml. The combined spinal-epidural anesthesia technique was performed using 1% ropivacaine hydrochloride and 2% lidocaine hydrochloride. Emergency medicines included atropine sulfate injection and ephedrine hydrochloride injection. In addition, flumazenil, a benzodiazepine antagonist, was at hand for urgency. For safety reasons, emergency endotracheal intubation devices and artificial ventilation devices were put on a standby.

Before putting patients to the operating room, we made sure that they had not taken any sedative or medication that may interfere with the surgery, anesthesia, or the investigational agent. All patients fasted for solids and water for at least 8 h preoperatively. After confirming that everything was set, patients were connected to the ECG monitor, their peripheral venous access built, venous catheter indwelled, and the compound sodium chloride administered intravenously. Vital signs, including blood pressure (BP), heart rate (HR), and blood oxygen saturation (SpO_2_) were observed. Patients inhaled room air throughout the process without additional oxygenation. After 15 min of observation, they received spinal-epidural anesthesia when vital signs were stable.

### Conduct of the study

Local anesthesia and puncture were performed at L3/4 in a lateral decubitus position by an anesthesiologist with 2-3 ml 2% lidocaine hydrochloride. A successful puncture was marked by the clear and free-flow cerebrospinal fluid (CSF). Then 3 ml 0.5% ropivacaine hydrochloride (diluted with patients’ own CSF) was injected. A catheter was placed in the epidural space with no medication being injected. Upon anesthesia completion, patients changed to a supine position, whose level of anesthesia was then assessed using the Modified Bromage Scale [14]. Then, they changed to a lithotomy position for surgical preparation when the anesthesia plane was between T6 and T8 after 15 min. Then patients were connected to BIS and PSI electrodes. Considering hair volume and other factors, sticking areas varied, mostly on patients’ forehead and temples. At the beginning of the operation, after confirming that patients were in a completely painless state, they were instructed to close their eyes for 1 min, and the BIS and PSI values recorded at this time were baseline levels [15]. An anesthesiologist injected 0.1 mg/kg of RT intravenously at a uniform speed within 1 min. BIS, PSI and MOAA/S score were recorded sequentially every minute. Then mean arterial pressure (MAP), HR, SpO_2_, pleth variability index (PVI) and other indicators of safety were recorded for a total of 10 min. Anesthesia for routine surgery was performed. Anesthesia-related adverse events were documented.

### Study parameters and study outcomes

The MOAA/S Scale [14] was evaluated after recording the BIS and PSI since this assessment could impact patients’ state of consciousness. In addition, MAP, HR, SpO_2_, PVI, liver and kidney function indicators including alanine transaminase (ALT), aspartate transaminase (AST), total bilirubin (TBil), serum creatinine (sCr), blood urea nitrogen (BUN), glomerular filtration rate (GFR) 24 h before and 24 h after surgery were also recorded to evaluate the safety of RT. Injection-site pain, nausea and vomiting, agitation, delirium and other adverse events were documented.

In our study, RT was used in elderly patients for supportive sedation. Primary outcomes were correlations between any two of the three: BIS, PSI, and MOAA/S scores. Secondary outcomes included vital signs (MAP, HR, SpO_2_, and PVI) and liver and kidney function parameters (ALT, AST, TBil, sCr, BUN, and GFR) for safety evaluation.

### Statistical analysis

Statistical analysis was performed using SPSS Statistics 25™ (SPSS Inc., Chicago, IL, U.S.A.). Pearson’s Correlation Coefficient (r) was calculated to assess correlations between BIS and PSI, and between BIS or PSI and MOAA/S Scale, respectively. Determination coefficients (r^2^ linear) were calculated by a linear regression model [15, 16]. Values were expressed as mean ± standard deviation (SD), mean (95% C.I.), or as numbers. A paired t-test was applied to test the changes of liver and kidney function before and after agent administration. Statistical significance was defined by a *P* value < 0.05. The area under the receiver operating characteristic (ROC) curve for each index was determined by plotting the sensitivity (fraction of MOAA/S score ≤ 3) against 1-specificity (fraction of MOAA/S scale > 3), which reflected the discriminating power of the indices. The area under the ROC curve summarizes the predictive power to achieve a high specificity at any given sensitivity. An area > 0.5 indicates that the measurement is predictive, and a measurement with 100% accuracy would have an area of 1.0 [15].

It was a single-arm study without randomization. The sample size was calculated using PASS 15™( NCSS, LLC. Kaysville, Utah, USA.) software. the power value (1-β) was set to 0.95 and the α value was set to 0.05. ρ_0_ ( baseline correlation) was set to 0. Based on the expected minimum r value of 0.3 obtained from the previous experiment, ρ_1_ ( alternative correlation) was set to 0.3. The final calculation shows that we need 138 pairs of data to perform our experiments. For each patient, 11 sets of data were recorded. In total, we collected 440 sets of data for statistical analysis. Therefore, we considered the sample size sufficient.

## Results

In total, 40 completed the visit 24 h before surgery, the anesthesia, and the surgery procedure. Demographic data are presented in Table 1.

**Table 1:** Demographic characteristics

Characteristics	*n* = 40
Age (year)	71.37 ± 4.66
Height (cm)	160.75 ± 6.37
Weight (kg)	57.98 ± 6.49
BMI (kg/m^2^)	22.39 ± 1.51

**Fig. 1:**
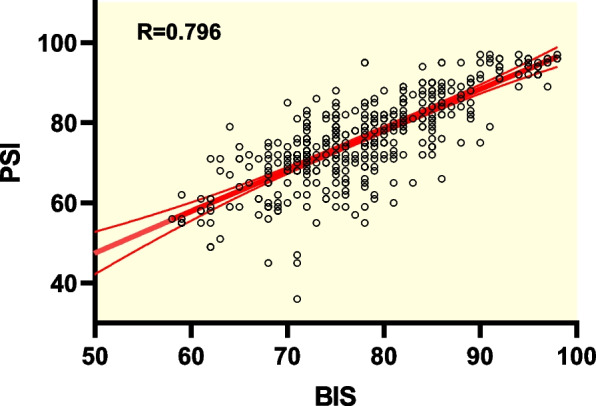
Scattergram of correlation between BIS and PSI. Regression line and 95% confidence interval. Correlation coefficient (*r* = 0.796)

**Fig. 2:**
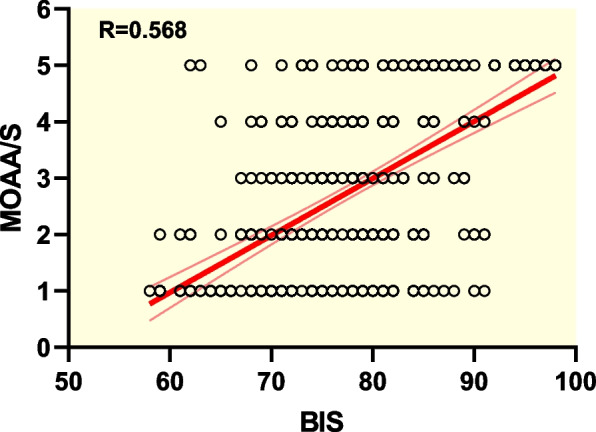
Scattergram of correlation between BIS and MOAA/S scale. Regression line and 95% confidence interval. Correlation coefficient (*r* = 0.568)

**Fig. 3:**
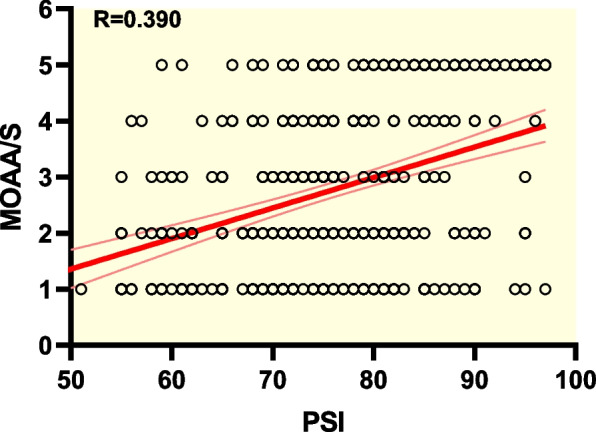
Scattergram of correlation between PSI and MOAA/S scale. Regression line and 95% confidence interval. Correlation coefficient (*r* = 0.390)

**Fig. 4:**
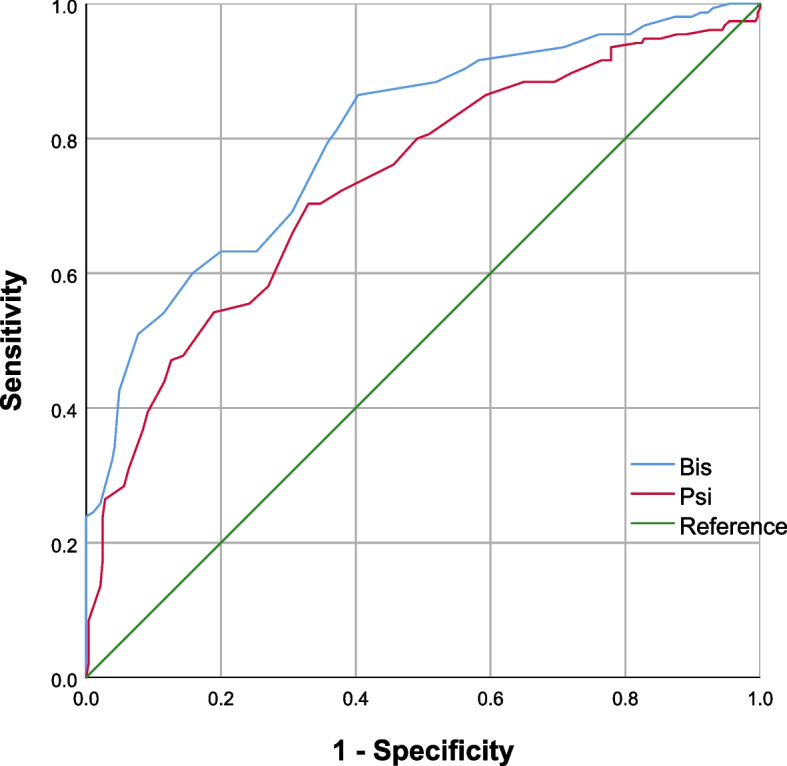
ROC curves for discrete threshold values of BIS and PSI. The area under the BSI curve was greater than that of the PSI curve (0.801 ± 0.022 vs. 0.734 ± 0.026)

**Fig. 5:**
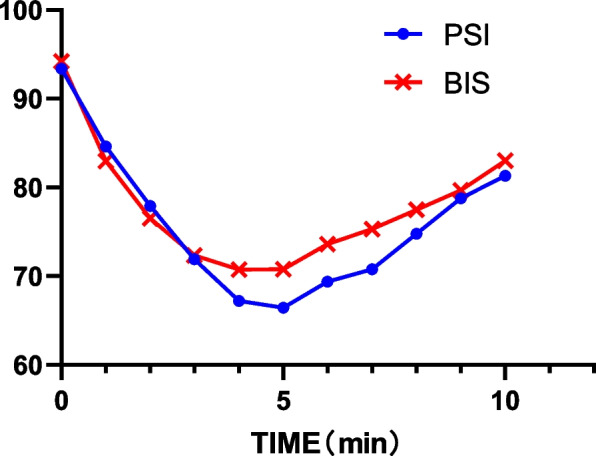
The trend of BIS and PSI over time

Shown in Fig. 1, based on Pearson’s correlation analysis, a significant (*P* < 0.01) correlation between BIS and PSI was found (*r* = 0.796). Significant associations between BIS and MOAA/S (*r* = 0.568, *P* < 0.01), and between PSI and MOAA/S (*r* = 0.390, *P* < 0.01) were also found, with scattergrams shown in Figs. 2 and 3, respectively. The areas under the ROC curves (Fig. 4) of BIS and PSI were 0.801 ± 0.022 and 0.734 ± 0.026, respectively, suggesting that both measures may predict patients’ state of consciousness. Figure 5 show trends in BIS and PSI over time. During our study, a MAP 20% less or greater than the baseline value, arrhythmia, and hypoxemia (SpO_2_ less than 90%) were not observed. Changes of liver and kidney function parameters 24 h before and 24 h after surgery are shown in Supplementary materials. No significant differences were observed in the levels of ALT and AST (*P* > 0.05). Nonetheless, levels of TBil changed significantly (*P* < 0.05). Significant improvements were seen in renal function parameters, including sCr, BUN, and GFR (*P* < 0.01).

## Discussion

In this single-arm, single-center, prospective clinical trial, we attempted to compare two different modalities of anesthesia depth monitoring (BIS and PSI) in patients undergoing supportive sedation with remimazolam tosilate for injection. Correlation analyses were also carried out between BIS or PSI and MOAA/S score, a commonly used clinical evaluation of sedation, to further verify the rigor of our results. Additionally, we evaluated the clinical safety of RT, a novel benzodiazepine, by evaluating commonly applied monitoring indicators and laboratory test parameters.

At present, there are a few studies exploring the correlation between BIS and PSI, and most of them were conducted in an intraoperative monitoring setting of general anesthesia with endotracheal intubation [15]. We believe that there are two confounders when using general anesthetics and sedatives in combination with opioids and other analgesics. First, different doses of analgesics will greatly impact patients’ state of consciousness [17]. Such evaluation is not based on a single component, and the mixed administration of various sedatives and analgesics may result in variations in sedation depth. Second, even if patients are under anesthesia, no intravenous analgesic can achieve complete painlessness. Ideally, the optimal assessment of such correlation would be in healthy volunteers using the investigational agent alone. The biggest difference between our study and previous ones lies in that we selected surgical patients under intraspinal anesthesia, relieving pain stress in patients, realizing monotherapy to minimize the effect of confounders, and thus maximizing the plausibility of our results.

Studies have shown that BIS monitoring is not sensitive to benzodiazepines compared to propofol [4]. It is not difficult to understand since BIS was originally developed for propofol, a classic sedative. However, with the progress of clinical practices and scientific research, the application of BIS in the intraoperative monitoring of benzodiazepines, represented by midazolam, has greatly reduced the dose of anesthetic [4, 18]. In real-world clinical settings, it is plausible to use BIS in monitoring benzodiazepine-assisted sedation. In contrast, the patented database of PSI does not rely on any anesthetics. Therefore, the design of associating the two monitoring methods used in assessing benzodiazepines intraoperatively may well solve clinical headaches and guide appropriate clinical use of anesthetic agents.

RT is a novel benzodiazepine used in sedation. With the rapid advancement of its clinical application, its indications have extended from intraoperative sedation in gastrointestinal endoscopies when first launched to general anesthesia for tracheal intubation [19–21]. With a rapid onset of action (within 1 min), it reaches the peak blood concentration within three minutes [11, 22]. Within 7 min or so, MOAA/S scores comes to 4 or above. As such, it enjoys a broad prospect in intraoperative supportive sedation. The new benzodiazepine is rapidly degraded in plasma by nonspecific cholinesterases and liver carboxyesterases. Cardiac and respiratory depression were rare in previous studies on RT in gastrointestinal endoscopies. As such, it can be safely used in elderly patients. By studying recommended doses used in clinical studies in multiple centers, combined with research conclusions of our hospital, we chose 0.1 kg/mg for this study to ensure sufficient anesthesia depth that can be observed, recorded and analyzed on the basis of intraoperative safety [10, 22–24]. Based on our observation, all patients achieved MOAA/S score ≥ 4 within 7 min of RT administration. At 10-min post administration, MOAA/S scores stood at 5 for most patients, interfering with statistical analyses to some extent. As such, we chose 10 min post-dosing as the endpoint of data collection.

From our results, there is a strong correlation between BIS and PSI for anesthesia depth monitoring, which is consistent with previous studies. we also considered that remimazolam is a short-acting sedative drug and clinical pharmacodynamic parameters may change rapidly, to more accurately reflect the objective clinical status, we introduced the MOAA/S score as a bridge to compare the sensitivity and accuracy of remimazolam sedation monitored by BIS and PSI. It is worth pointing out that although PSI is anesthetic-independent, changes of PSI and BIS with the depth of anesthesia are similar. Comparing the correlation between BIS and MOAA/S and that between PSI and MOAA/S, we found that the former showed a stronger association (r_bis_ > r_psi_). We can therefore conclude that BIS is superior to PSI in assessing intraoperative supportive sedation with RT. According to Professor Chen from the Department of Anesthesiology and Pain Management, University of Texas Southwestern Medical Center at Dallas, after adding the anesthetic, the value of PSI was always lower than that BIS, and with the recovery of patient orientation, the value of PSI never returned to the baseline level before induction. Based on these results, the team conclude that there are differences in the sensitivity of the two methods to residual (subhypnotic) levels of anesthetics and/or intraoperative “shifts” in PSI values [15]. We observed a similar pattern in our study. This phenomenon was more pronounced due to the short action duration of RT. This partially explains why PSI values remained low while MOAA/S scores recovered to 5 and why r values were impacted. Contrary to the previous study, however, the areas under our ROC curves suggest that BIS may have greater sensitivity and/or specificity for changes in consciousness levels, mainly led by different anesthesia applied in the two studies. It is worth pointing out that our use of a MOAA/s score of 3 as a threshold is based on our previous study of ED_95_ versus ED_50_ for moderate sedation with remimazolam [25], combined with previous studies of relevant doses of other sedative drugs during moderate sedation. Changes in the patient’s response to subjective stimulation by the anesthesiologist can be observed visually when the MOAA/S value is at 3, mainly in response to only after name is called loudly and/or repeatedly. Therefore, we used the MOAA/S score of 3 as a junction to perform ROC curves to analyze the sensitivity of sedation monitoring instruments. We did not use general anesthesia for tracheal intubation, thus the losing and recovering of consciousness was a relatively simple process in our study, without confounders such as additional agents and tracheal intubation stimulation. In addition, we only used benzodiazepines for intraoperative supportive sedation, which is one of our uniqueness.

We compared changes of vital signs and liver and kidney functions before and after surgery. Results showed that vital signs were stable, which is consistent with previous studies [22]. RT is superior in maintaining cardiopulmonary stability compared with other anesthetics and sedatives including propofol. However, it should be noted that we did not monitor arterial blood gas and end-respiratory carbon dioxide levels, nor did we perform endotracheal intubation or arterial puncture. As such, we could not rigorously assess the effect of RT on ventilation function but attempt to do so in future studies. PVI, commonly used in intensive care unit (ICU), is a non-invasive method to comprehensively assess patients’ cardiopulmonary function and circulatory function [25, 26]. In our study, PVI showed an upward trend after RT administration, but with a short duration and a small size of effect. The safety of RT administered by continuous infusion needs to be verified by follow-up studies.

Metabolized by tissue esterases and liver carboxyesterases, theoretically, RT would not increase hepatic and renal burden [11]. However, according to preliminary results of our study and the manufacturer’s instructions, an elevated level of blood bilirubin may occur in some patients. RT is catalyzed by CES1 in the liver to produce an inactive metabolite, CNS7054. Based on an in vitro hepatocyte exposure study, no deleterious changes were observed in hepatocytes, although the metabolic pathway of RT is partly through hepatic carboxylesterase metabolism [27]. In our study, no increase above the upper limit of normal was observed. In addition, patients used antibiotics and other surgical-related medications perioperatively, we thus cannot rule out possible drug-drug interactions which may affect bilirubin metabolism. Renal function tends to be improved in our study. However, since electro-prostatectomy can largely relieve the symptoms of urinary system obstruction, we thus cannot attribute such renal effect to RT. Based on our results, RT is safe and controllable for intraoperative sedation.

There are limitations. First, it is a single-center, single-arm study. Even though we collected 440 sets of data, which were sufficient for Pearson’s correlation analysis, considering patient heterogeneity, a larger sample size may increase the accuracy. Second, some studies have pointed out that compared with BIS monitoring, electro-resection produces less effect on PSI. In our trial, the operating time is relatively short, and no strong evidence confirms this conclusion. Third, the clinical application of RT needs to be validated using multiple anesthesiologic approaches and laboratory test parameters. Further, Because of the rapidly changing pharmacokinetics of remimazolam, the state of consciousness of subjects during a single dose of remimazolam is also changing rapidly. Therefore there may be a lag in the identification of sedation monitoring devices. The purpose of this study focused on pharmacodynamics in clinical applications with a view to finding a more sensitive and accurate monitoring index in clinical applications, so no blood was collected to determine blood concentrations. Therefore, it is difficult to dynamically assess the pharmacokinetic parameters during single-dose sedation of remimazolam in our trial design, and further validation in subsequent continuous sedation trials is expected.

## Conclusions

BIS and PSI are strongly associated for monitoring the sedation of RT intraoperatively. Both can accurately reflect the trend of sedation depth. According to correlation analyses with MOSS/S scale and ROC curves, BIS is more accurate than PSI during such intraoperative monitoring. In addition, RT can be safely used in elderly patients under intraspinal anesthesia for supportive sedation, with stable vital signs and sound kidney and liver safety profiles.

## Supplementary Information

**Additional file 1: Supplementary Figure 1.** Intraoperative MAP changes. **Supplementary Figure 2.** Intraoperative HR changes. **Supplementary Figure 3.** Intraoperative SpO2 changes. **Supplementary Figure 4.** Intraoperative PVI changes. **Supplementary Table 1.** Modified Bromage Scale. **Supplementary Table 2.** Modified Observer’s Assessment of Alertness/Sedation (MOAA/S) scale. **Supplementary Table 3.** Laboratory tests of the patient's liver and kidney function.

## Data Availability

The data that support the findings of this study are available from the corresponding author upon reasonable request.

## Abbreviations

RT: Remimazolam tosilate
BIS: Bispectral Index
PSI: Patient State Index
MOAA/S: Modified Observer’s Assessment of Alertness/Sedation
ASA: American Society of Anesthesiologist
BMI: Body mass index
CSF: Cerebrospinal fluid
ECG: Electrocardiogram
NIBP: Noninvasive blood pressure
SBP: Systolic blood pressure
DBP: Diastolic blood pressure
SpO_2_: Blood oxygen saturation
RR: Respiratory rate
HR: Heart rate
