# Supplementary material for: Comparison of bispectral index and patient state index as measures of sedation depth during surgeries using remimazolam tosilate
Source: BMC Anesthesiol. 2023 Jun 15;23:208. doi: 10.1186/s12871-023-02172-3 (PMC10268360; doi:10.1186/s12871-023-02172-3)
Supplement: Supplementary file 1 — Additional file 1: Supplementary Figure 1. Intraoperative MAP changes. Supplementary Figure 2. Intraoperative HR changes. Supplementary Figure 3. Intraoperative SpO2 changes. Supplementary Figure 4. Intraoperative PVI changes. Supplementary Table 1. Modified Bromage Scale. Supplementary Table 2. Modified Observer’s Assessment of Alertness/Sedation (MOAA/S) scale. Supplementary Table 3. Laboratory tests of the patient's liver and kidney function. [file 12871_2023_2172_MOESM1_ESM.zip › Supplementary Table 2.pdf]

Scale	MOAA/S Scale
0	Does not respond to painful trapezius squeeze
1	Responds only after painful trapezius squeeze
2	Responds only after mild prodding or shaking
3	Responds only after name is called loudly and/or repeatedly
4	Lethargic response to name spoken in normal tone
5 (alert)	Responds readily to name spoken in normal tone

Supplementary Table 2. Modified Observer’s Assessment of Alertness/Sedation (MOAA/S) scale
