# Supplementary material for: Comparison of bispectral index and patient state index as measures of sedation depth during surgeries using remimazolam tosilate
Source: BMC Anesthesiol. 2023 Jun 15;23:208. doi: 10.1186/s12871-023-02172-3 (PMC10268360; doi:10.1186/s12871-023-02172-3)
Supplement: Supplementary file 1 — Additional file 1: Supplementary Figure 1. Intraoperative MAP changes. Supplementary Figure 2. Intraoperative HR changes. Supplementary Figure 3. Intraoperative SpO2 changes. Supplementary Figure 4. Intraoperative PVI changes. Supplementary Table 1. Modified Bromage Scale. Supplementary Table 2. Modified Observer’s Assessment of Alertness/Sedation (MOAA/S) scale. Supplementary Table 3. Laboratory tests of the patient's liver and kidney function. [file 12871_2023_2172_MOESM1_ESM.zip › Supplementary Table 3.pdf]

**characteristics**	24 hours before surgery (n=40)	24 hours after surgery (n=40)	*P* value
ALT (u/L)	18.10 ± 4.97	18.08 ± 5.72	*P = 0.975*
AST (u/L)	18.83 ± 4.89	19.73 ± 4.91	*P = 0.358*
TBil (μ mol/L)	10.34 ± 6.16	12.03 ± 5.25	*P= 0.008*
sCr (μ mol/L)	79.43 ± 26.31	71.80 ± 22.93	*P = 0.001*
BUN (mmol/L)	5.85 ± 1.75	4.83 ± 1.71	*P < 0.001*
GFR(ml/min)	82.73 ± 29.88	125.23 ± 60.02	*P < 0.001*

Supplementary Table 3. Laboratory tests of the patient's liver and kidney function
